# Systems analysis of transcriptome data provides new hypotheses about *Arabidopsis* root response to nitrate treatments

**DOI:** 10.3389/fpls.2014.00022

**Published:** 2014-02-07

**Authors:** Javier Canales, Tomás C. Moyano, Eva Villarroel, Rodrigo A. Gutiérrez

**Affiliations:** Department of Molecular Genetics and Microbiology, Faculty of Biological Sciences, FONDAP Center for Genome Regulation, Millennium Nucleus Center for Plant Functional Genomics, Pontifical Catholic University of ChileSantiago, Chile

**Keywords:** meta-analysis, root hairs, nitrate, systems biology, gene co-expression analysis, transcription factors, Gene Ontology (GO)

## Abstract

Nitrogen (N) is an essential macronutrient for plant growth and development. Plants adapt to changes in N availability partly by changes in global gene expression. We integrated publicly available root microarray data under contrasting nitrate conditions to identify new genes and functions important for adaptive nitrate responses in *Arabidopsis thaliana* roots. Overall, more than 2000 genes exhibited changes in expression in response to nitrate treatments in *Arabidopsis thaliana* root organs. Global regulation of gene expression by nitrate depends largely on the experimental context. However, despite significant differences from experiment to experiment in the identity of regulated genes, there is a robust nitrate response of specific biological functions. Integrative gene network analysis uncovered relationships between nitrate-responsive genes and 11 highly co-expressed gene clusters (modules). Four of these gene network modules have robust nitrate responsive functions such as transport, signaling, and metabolism. Network analysis hypothesized G2-like transcription factors are key regulatory factors controlling transport and signaling functions. Our meta-analysis highlights the role of biological processes not studied before in the context of the nitrate response such as root hair development and provides testable hypothesis to advance our understanding of nitrate responses in plants.

## Introduction

Nitrogen (N) is a constituent of nucleic acids, amino acids, chlorophyll, and phytohormones among many other biomolecules. N is quantitatively the most abundant element in plant tissues after C, H, and O, representing up to 5% of dry weight (Miller and Cramer, 2005). However, biologically available N is often in short supply in natural as well as agricultural systems, limiting plant growth and development. Understanding the molecular mechanisms underlying plant N responses is an important issue for plant biology as well as agriculture. Transcriptomics approaches have been used extensively to investigate these mechanisms. Transcriptomic studies have evaluated different aspects of the plant nitrogen response, such as the effect of N source (e.g., Wang et al., 2007; Patterson et al., 2010), time after N treatments (e.g., Krouk et al., 2010a), N concentration (e.g., Wang et al., 2007), tissue type (e.g., Wang et al., 2003), and cell type (e.g., Gifford et al., 2008). These studies have identified a myriad of genes and functions modulated by N nutrient/metabolites such as uptake, transport and metabolism (reviewed in Vidal and Gutiérrez, 2008; Alvarez et al., 2012).

The nitrate signaling pathway is still poorly understood with only a handful regulatory factors modulating gene expression at different levels identified. Plants perceive changes in the concentration of nitrate by specific receptors. It has been established that the dual-affinity nitrate transporter NRT1.1 is able to function as nitrate sensor in *Arabidopsis thaliana* (Ho et al., 2009). Additional regulatory factors include protein kinases from the CIPK family. For example, loss of function of *CIPK8* gene reduces by 40–65% the expression of several primary response genes such as *NRT1.1*, *NIA1*, or *GLN2* (Hu et al., 2009). Another level of control of gene expression by nitrate is carried out by transcription factors. Only a few transcription factors directly implicated in regulating nitrate responses have been characterized to date (Gutiérrez, 2012). Some of these transcription factors are important for the control of nitrate assimilation, while others have been involved in modulation of root system architecture. The first transcription factor identified in the N response was a MADS box transcription factor called *ANR1* (Zhang et al., 1999). *ANR1* is involved in the control of lateral root growth in response to localized nitrate supply. Recent studies indicate NLPs are also important transcription factors in the nitrate response because they regulate many known nitrate signaling and assimilation genes (Konishi and Yanagisawa, 2013; Marchive et al., 2013). Additional transcription factors known to regulate N responses include members of the LBD family (Rubin et al., 2009); *HY5*, which is related to phytochrome-mediated effects on enzymes involved in nitrogen assimilation (Lillo, 2008); and a nitrate-induced *NIGT1* member of the GARP family, which has been suggested to be involved in the control of nitrate utilization (Sawaki et al., 2013). Recently, *NAC4* was found to be a key regulatory element controlling a nitrate-responsive network and lateral root development in *Arabidopsis* (Vidal et al., 2013). However, nitrate regulation of other important features of root system architecture such as primary root growth and root hair development have not yet been explored in depth (Forde and Walch-Liu, 2009). Several studies reported root hairs are important for nutrient uptake, including nitrogen uptake (Libault et al., 2010). Wang et al. (2002) observed root hair growth in response to nitrate treatment in rice. In the case of other macronutrients, such as phosphate, many genes involved in root morphological response to nutrient availability (Niu et al., 2013), including root hair responses (Lin et al., 2011a,b), have been identified. However, the role of root hairs and root hair development in nitrate responses has not been addressed.

The development of high-throughput technologies enabled identification of more than 2000 genes with changes in gene expression in response to various N treatments (Krouk et al., 2010b). However, functional characterization of these genes is long and laborious and lags far behind global gene expression studies. In order to aid prioritizing, we integrated publicly available root nitrate transcriptome data obtained under various experimental conditions. The analysis presented here (i) identifies the most consistent genes and biological functions regulated in response to nitrate treatments across multiple experiments (ii) highlights important biological process associated with nitrate root responses that have not been previously addressed and (iii) proposes key regulatory factors controlling major gene network modules. Our integrated systems analysis provides concrete testable hypothesis to advance our understanding of plant root nitrate responses.

## Methods

### Microarray data collection and preprocessing

Microarray data used in this work was obtained from experiments published in: (Wang et al., 2003, 2004, 2007; Gutiérrez et al., 2007a; Gifford et al., 2008; Hu et al., 2009; Krouk et al., 2010a; Patterson et al., 2010; Ruffel et al., 2011; Vidal et al., 2013 and Álvarez et al., submitted.) CEL files of these experiments are available in the public microarrays databases GEO, EBI, or NASC. Accession numbers for each data set are indicated in Table 1. Background correction and normalization of the raw data sets was performed using Robust MultiChip Analysis (RMA) implemented in “affy” R package (Gautier et al., 2004).

**Table 1:** **Summary of microarray datasets and experimental parameters**.

**References**	**Accession number**	**Database**	**Number of arrays selected**	**Name in this paper**	**Nitrate concentration (mM)**	**Time treatment (min)**	**Tissue**	**Growth stage**	**Growth condition**
Wang et al., 2003	NASCARRAYS-479	NASC Arrays	4	1	0.25	20	Whole roots	Seedling	Hydroponic
Wang et al., 2004	NASCARRAYS-480	NASC Arrays	4	2	5	120	Whole roots	Seedling	Hydroponic
Gutiérrez et al., 2007a	E-MEXP-828	Array Express	8	3	5	480	Whole roots	Adult	Hydroponic
				4	10	480	Whole roots	Adult	Hydroponic
				5	15	480	Whole roots	Adult	Hydroponic
Gifford et al., 2008	GSE7631	GEO	42	6	5	120	Lateral root cap	Seedling	Hydroponic
				7	5	120	Epidermis + Cortex	Seedling	Hydroponic
				8	5	120	Endodermis + Pericycle	Seedling	Hydroponic
				9	5	120	Pericycle	Seedling	Hydroponic
				10	5	120	Stele	Seedling	Hydroponic
				11	5	120	Whole roots	Seedling	Hydroponic
				12	5	210	Whole roots	Seedling	Hydroponic
Krouk et al., 2010a	GSE20044	GEO	24	13	1	3	Whole roots	Seedling	Hydroponic
				14	1	6	Whole roots	Seedling	Hydroponic
				15	1	9	Whole roots	Seedling	Hydroponic
				16	1	12	Whole roots	Seedling	Hydroponic
				17	1	15	Whole roots	Seedling	Hydroponic
				18	1	20	Whole roots	Seedling	Hydroponic
Hu et al., 2009	GSE9148	GEO	6	19	25	30	Whole roots	Seedling	Hydroponic
Ruffel et al., 2011	GSE22966	GEO	18	20	5	120	Whole roots	Seedling	Agar plates
				21	5	480	Whole roots	Seedling	Agar plates
				22	5	2880	Whole roots	Seedling	Agar plates
Patterson et al., 2010	GSE29589	GEO	9	23	1	90	Whole roots	Seedling	Hydroponic
				24	1	480	Whole roots	Seedling	Hydroponic
Vidal et al., 2013	GSE35544	GEO	6	25	5	120	Whole roots	Seedling	Hydroponic
Álvarez et al., submitted	GSE43011	GEO	6	26	5	120	Whole roots	Seedling	Hydroponic
Wang et al., 2007	NASCARRAYS-481	NASCArrays	4	27	0.25	20	Whole roots	Seedling	Hydroponic

### Identification of differentially expressed genes

The non-parametric RankProduct method (Breitling et al., 2004) was used to identify differentially expressed genes between treatment and control conditions (potassium nitrate vs. potassium chloride), as this method has been shown to be robust in analyzing microarray data that comprise small numbers of replicates (Hong and Breitling, 2008). A gene was considered statistically significant if its false discovery rate (FDR) adjusted *p*-value was equal or smaller than 0.05.

### Network construction and coexpression module detection

A weighted gene coexpression network was constructed using the WGCNA R package version 1.27.1 (Langfelder and Horvath, 2008) with differentially expressed genes. First, the Pearson correlation matrix was weighted by raising it to a power (β). To choose the appropriate power, the network topology for various soft-thresholding powers was evaluated using pickSoftThreshold function and β = 7 was chosen because this ensured an approximate scale-free topology of the resulting network as previously described (Zhang and Horvath, 2005). Next, the pairwise measure of gene coexpression of the resulting weighted network was transformed into a topological overlap (TO) similarity measure, which is a robust measure of pairwise interconnectedness (Yip and Horvath, 2007). A TO similarity measure between two genes (*ij*) is defined as: ${\text{TO}}_{ij}=\frac{\sum_{u}a_{iu}a_{uj}+a_{ij}}{\min(k_{i},\;k_{j})+1-a_{ij}}$ where *k_i_* = ∑_*u*_*a_iu_* was the node connectivity and *a* is the network adjacency. Finally, TO similarity measure coupled with average linkage hierarchical clustering was performed for module detection using the Dynamic Tree Cut algorithm (Langfelder et al., 2008). The coexpression network was visualized using Cytoscape v 3.0 (File S1) and analyzed using the NetworkAnalyser plugin (Doncheva et al., 2012). In order to simplify the display of the network and to focus on relevant relationships, only edges in this network of the corresponding TO similarity measure above a threshold of 0.10 are shown in Figure 3. Further, NetworkAnalyser plugin was used to assess which genes in the network form hubs. VirtualPlant platform (Katari et al., 2010) was used to generate a subnetwork of transcription factors and putative targets taking into account the TO similarity measure and over-represented transcription factors binding sites.

### Functional enrichment analysis

Fisher's exact test was performed for declaring a GO (Gene Ontology) category as significantly over-represented (Benjamini–Yekutieli method for controlling FDR, adjusted *p*-value < 0.05) using PlantGSEA toolkit (Yi et al., 2013). GO level was defined as the number of edges in the shortest path connecting a node to the root node, this information was calculated using SQL queries on the GO database. To determine protein sequence similarity for genes associated to GO terms, we performed pair-wise BLASTP sequence comparisons. We only analyzed alignments with an *E*-value smaller or equal than 10^−10^. To compare the results we used protein sequence similarity scores normalized by query length.

## Results and discussion

### Members of G2-like and LBD transcription factor families are the most consistently regulated genes in response to nitrate treatments

**Figure 1:**
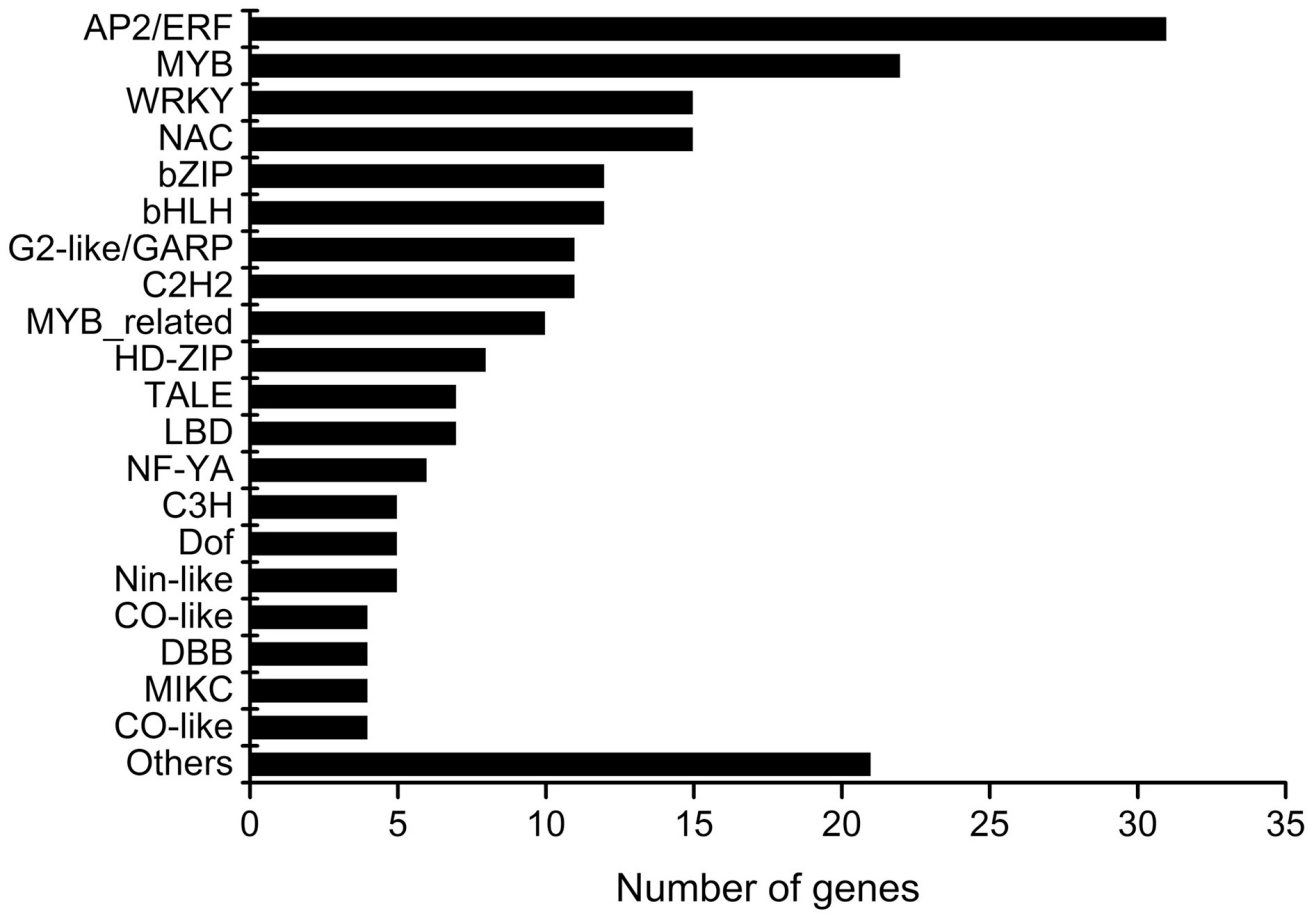
**Distribution of the 219 nitrate-responsive transcription factors according to family**. Transcription factors were classify following PlantTFDB2.0 database annotation (Zhang et al., 2011).

Meta-analysis of microarray data has been used to define robust sets of regulated genes (e.g., Gutiérrez et al., 2007b; Bhargava et al., 2013). To gain new insights into the plant N response we carried out a meta-analysis of transcriptome data based on the following criteria: First, we focused on experiments performed with nitrate as the only experimental factor. Nitrate is one of most important nitrogen sources in agricultural soils (Glass, 2009) and it can act as a signal to regulate global gene expression as well as different aspects of plant growth and development (Wang et al., 2004). Second, we focused on transcriptome studies carried out in root organs because it is the first organ encountering nitrate and initiates plant responses (Wang et al., 2003) and it is the organ for which more data sets are available. Finally, we focused on studies with wild-type genetic background and Affymetrix microarray platform to avoid cross-platform variation as well as unbalanced data sets. Using these criteria, 27 experimental datasets corresponding to 131 ATH1 Affymetrix genechip hybridizations were selected for further analysis (Table 1). To focus on relevant N-responsive genes, RMA normalization (Irizarry et al., 2003) was performed and Rank Products (Breitling et al., 2004) was used to identify genes that exhibited expression differences between nitrate treatment and control (KCl) samples in each experiment. A total of 2286 genes were identified as differentially expressed in at least one experiment, with a maximum FDR of 5% (Table S1). Next, we ranked differentially expressed genes by the number of experiments in which they were regulated. The top 50 genes according to this criterion are shown in Table 2. No gene was differentially expressed in response to nitrate treatments in all experiments. The expression of all genes in Table 2 was induced by nitrate treatments in at least 40% of the experiments analyzed, with a maximum of 75%. The majority of these core nitrate-response genes were upregulated. Starvation pre-treatment causes a decrease in expression levels of many genes related to nitrate assimilation, signaling, and transport. An increase in the expression of many genes is therefore expected after the nitrate treatment. Interestingly, the top 5 most consistent genes found in our analysis code for transcription factors. *HRS1* (AT1G13300), a G2-like transcription factor, was statistically induced in 20 out of 27 experiments (Table 2). *HRS1* is expressed in root hairs and is induced under low phosphate concentration (Liu et al., 2009). *HRS1* may be involved in modulation of primary root and root hair growth in response to phosphate starvation (Liu et al., 2009). Our results suggest that in addition to phosphate, *HRS1* is a regulator of root growth in response to nitrogen availability. Three other members of this transcription factor family respond to nitrate in multiple experiments: AT3G25790, AT1G68670, and AT1G25550. AT1G68670 is a direct target of *SHORT ROOT* (*SHR*) (Cui et al., 2011), a key regulator of root growth and development in *Arabidopsis*. Moreover, *SHR* regulates cytokinin homeostasis by directly controlling transcription of *CYTOKININ OXIDASE 3* gene (Cui et al., 2011). Several studies indicate nitrate induces the expression of cytokinin biosynthesis genes resulting in cytokinin accumulation in response to nitrate (Kiba et al., 2011). These observations suggest G2-like transcription factor (AT1G68670) is part of the *SHR* regulatory network modulating root development and cytokinin levels. The second G2-like family gene AT1G25550, was found to bind the *E2Fa* promoter. *E2Fa* is an essential transcription factor in *A. thaliana* that regulates asymmetric cell division marking lateral root initiation (Berckmans et al., 2011). These findings suggest that G2-like transcription factors may be important regulators of root morphology in response to nitrate availability. Other transcription factors that respond to nitrate in a large number of experiments (70%) are *LBD37* and *LBD39*. Transcription factors of the LBD family have been shown to have important roles in regulation of anthocyanin synthesis and nitrogen metabolism by repressing transcripts that are critical for nitrate transport and assimilation (Rubin et al., 2009). Additional transcription factors involved in *Arabidopsis* nitrate root responses include other previously characterized NAC and NLP transcription factors as well as a list of new candidates. Figure 1 shows the most represented transcription factor families in decreasing order. The most represented families are ERF/AP2 (31) and WRKY (15) transcription factors, with an overrepresentation of nitrate responsive genes as expected from their gene family size. These two transcription factor families are involved in plant development and responses to biotic and abiotic stress (Rushton et al., 2010; Mizoi et al., 2012). However, their role in nitrate responses is not clear yet. Another family of transcription factors with many members are MYB-type transcription factors (22). *MYB75* is known for its role in response to nitrogen limitation (Lea et al., 2007). In other plant species, MYB proteins are known to control expression of genes coding for important nitrogen assimilation enzymes, such as glutamine synthetase (Gómez-Maldonado et al., 2004) and asparagine synthetase (Canales et al., 2012). These results point toward specific transcription factors as candidate regulators controlling hallmark responses to nitrate treatments such as metabolism and root system architecture.

**Table 2:** **Ranking of the top 50 most consistent genes in response to nitrate**.

**AGI locus**	**Description**	**Total**	**Upregulated**	**Downregulated**
At1g13300	G2-like transcription factor family protein (*HRS1*)	20	20	0
At5g67420	LOB domain-containing protein 37 (*LBD37*)	19	19	0
At4g37540	LOB domain-containing protein 39 (*LBD39*)	19	19	0
At3g25790	G2-like transcription factor family protein	19	19	0
At1g49500	G2-like transcription factor family protein	18	18	0
At1g24280	Glucose-6-phosphate dehydrogenase 3 (*G6PD3*)	17	17	0
At5g01740	Nuclear transport factor 2 family protein (*NTF2*)	16	16	0
At3g48360	BTB and TAZ domain protein 2 (*BT2*)	16	16	0
At1g25550	G2-like transcription factor family protein	16	16	0
At1g80380	P-loop containing nucleoside triphosphate hydrolases	16	16	0
At5g40850	Urophorphyrin methylase 1 (*UPM1*)	15	15	0
At5g10210	Unknown	15	15	0
At1g77760	Nitrate reductase 1 (*NIA1*)	15	15	0
At1g78050	Phosphoglycerate/bisphosphoglycerate mutase (*PGM*)	15	15	0
At2g15620	Nitrite reductase 1 (*NIR)*	15	15	0
At2g26980	CBL-interacting protein kinase 3 (*CIPK3*)	15	15	0
At4g02380	Senescence-associated gene 21 (*SAG21*)	14	14	0
At3g07350	Unknown	14	14	0
At5g19970	Unknown	13	13	0
At5g62720	Integral membrane HPP family protein	13	13	0
At5g09800	ARM repeat superfamily protein	13	13	0
At3g49940	LOB domain-containing protein 38 (*LBD38*)	13	13	0
At3g16560	Protein phosphatase 2C family protein	13	13	0
At1g30510	Root FNR 2 (*RFNR2*)	13	13	0
At2g16060	Hemoglobin 1 (*AHB1*)	13	12	1
At5g41670	6-phosphogluconate dehydrogenase family protein	12	12	0
At5g19120	Eukaryotic aspartyl protease family protein	12	12	0
At4g25835	P-loop containing nucleoside triphosphate hydrolases	12	12	0
At4g05390	Root FNR 1 (*RFNR1*)	12	12	0
At1g49860	Glutathione S-transferase 14 (*GSTF14*)	12	12	0
At1g68880	Basic leucine-zipper 8 (*bZIP*)	12	11	1
At1g16170	Unknown	12	12	0
At2g48080	2OG-Fe(II) oxygenase family protein	12	12	0
At2g30040	Mitogen-activated protein kinase kinase kinase 14 (*MAPKKK14*)	12	12	0
At5g13110	Glucose-6-phosphate dehydrogenase 2 (*G6PD2*)	11	11	0
At5g45340	Cytochrome P450 (C*YP707A3*)	11	11	0
At3g62930	Thioredoxin superfamily protein	11	11	0
At3g47980	Integral membrane HPP family protein	11	11	0
At4g37610	BTB and TAZ domain protein 5 (*BT5*)	11	11	0
At4g32950	Protein phosphatase 2C family protein	11	9	2
At4g18340	Glycosyl hydrolase superfamily protein	11	11	0
At1g78090	Trehalose-6-phosphate phosphatase (*TPPB*)	11	11	0
At1g32920	Unknown	11	11	0
At1g68670	G2-like transcription factor family protein	11	11	0
At5g15830	Basic leucine-zipper 3 (*bZIP3)*	10	10	0
At5g14760	L-aspartate oxidase (*AO*)	10	9	1
At5g04950	Nicotianamine synthase 1 (*NAS1*)	10	10	0
At3g60750	Transketolase	10	10	0
At3g02850	STELAR K+ outward rectifier (*SKOR*)	10	10	0
At1g08090	Nitrate transporter 2.1 (*NRT2.1)*	10	10	0

*Total number of experiments in which each gene was regulated as well as if this regulation was up or downregulated are indicated*.

### Nitrate regulates a core set of biological functions regardless of the experimental context

**Figure 2:**
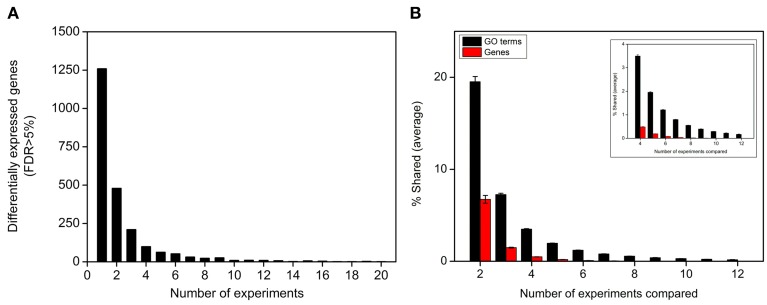
**Biological functions are more robust than gene identities in the nitrate response. (A)** Histogram of 2286 significantly nitrate responsive genes (rankproduct, FDR < 5%) vs. the number of experiments in which were regulated. **(B)** Histogram of the average percentage of elements (over-represented GO terms or genes) shared between different number of experiments. Inset shows the same graph but starting from 4 experiments.

As shown in Figure 2A, ~60% of differentially expressed genes were regulated by nitrate in only one experiment, consistent with the idea that nitrate regulation of gene expression depends largely on the experimental context (Gutiérrez et al., 2007b; Krouk et al., 2010b). However, analysis of regulated biological functions showed that responses at the functional level are more robust from experiment to experiment as compared to genes (Figure 2B). The average number of genes shared between any two experiments is 6.7%, while 19.5% of overrepresented GO terms (FDR < 0.05) are shared in the same two experiments (Figure 2B). This difference in the percentage of shared genes vs. GO terms increases with the number of experiments compared. For example, the number of GO terms shared between any five experiments is 10 times higher than the number of shared genes. To evaluate whether this difference between intersection of genes and GO terms was biological or an artifact due to the nature of the gene to GO association data we carried out three control experiments: (1) Conservation of GO terms could be explained by genes annotated to very general GO term categories increasing the chance of intersection at the GO term level. To address this potential issue, we compared the distribution of total and shared over-represented GO terms between any combination of two experiments. As shown in Figure S1A, the distribution of levels is similar for total and shared GO terms indicating differentially expressed genes are not biased toward general GO term categories. (2) Nitrate-responsive genes identified here may have many GO annotations, thus, explaining increased overlap in GO terms. To rule out this possibility, we analyzed the number of GO terms associated with the list of 2286 nitrate responsive genes used in this study and compared with the GO annotations found in 1000 lists of 2286 randomly selected genes represented in the ATH1 Affymetrix Gene Chip. As shown in Figure S1B, the average number of GO terms and their distribution are very similar in both cases. This result indicates nitrate responsive genes have no more annotations than a random set of genes of the same size. (3) If the genes are shared when comparing experiments the GO terms will be shared as well. However, GO terms shared between any two experiments contained on average only 22.4% of the same genes (Figure S2). This result indicates most of the genes contributing to over-represented GO terms are different in each experiment.

A prediction of our hypothesis is that different members of the same gene family should be found contributing to shared GO terms between different experiments. To systematically test this hypothesis, we performed pair-wise comparisons of protein sequences for all genes annotated to shared GO terms between any two experiments. We then compared the distribution of protein sequence similarities for all pair-wise comparisons between genes contributing to a shared GO term in our data set vs. all genes annotated to that GO term. We found shared GO terms (74.5%) have more pairs of similar protein sequences coming from different experiments than expected by chance (α < 0.05). For example, *NRT2.2* gene was found differentially expressed in experiment 21 and *NRT2.5* was found differentially expressed in experiment 23. Both *NRT2.2* and *NRT2.5* are annotated to the shared GO term “Transport (GO:0006810).” Similarly, the shared GO term “Response to Carbohydrate (GO:0009743)” contains *GLN1;2* and *GLN1;1*, each regulated in different experiments.

The easiest interpretation of these results is that nitrate responses at the biological function level are more robust to experimental context than genes. This phenomenon could be explained by functional redundancy of different genetic components, a feature that is common to biological networks and has been proposed as a mechanism for robustness toward stochastic fluctuations (Whitacre, 2012). A similar idea is the degeneracy concept proposed by Edelman and Gally (2001), which defines the property whereby structurally different elements may perform the same or similar functions. This feature has been attributed not only to gene networks but also to neural networks and evolution (Edelman and Gally, 2001). This phenomenon may be particularly relevant in plants, where increased gene family sizes may provide higher adaptive capacity to environmental perturbations.

### Robust nitrate responsive biological processes highlight new nitrate controlled developmental processes

Which biological functions are most relevant for nitrate responses in roots? To answer this question, we ranked GO terms by the number of experiments in which they were present based on regulated genes with the corresponding annotation. In order to focus on specific functions, we only considered GO terms at level 7 and 8 and removed redundant terms using the REVIGO tool (Supek et al., 2011). Table 3 shows the list of the most consistent biological functions. In contrast to genes, the most consistent GO terms appear regulated in ~90% of the experiments analyzed. The most consistent biological functions are those related to nitrate transport and carbon metabolism. We also found categories associated with root morphogenesis that have not been studied in the context of nitrate responses such as trichoblast differentiation (GO:0010054, 64 genes). Trichoblasts are a subset of specialized epidermal cells from which root hairs emerge. These specialized cells play an important role in the uptake of water and nutrients by increasing root absorption surface (Gilroy and Jones, 2001). Phytohormones are important regulators of root hair growth and development. It has been reported that auxin and ethylene promote root hair elongation and growth (Muday et al., 2012). Interestingly, biological processes that are statistically enriched in response to nitrate (Table S2) include auxin biosynthesis (GO:0009851, 32 genes, *p*-value = 1.25 × 10^−7^). Genes associated with this function include several genes from the tryptophan-dependent auxin biosynthetic pathway (Mano and Nemoto, 2012) such as *TAR2* (Stepanova et al., 2008), *CYP79B2*, and *CYP79B3* (Zhao et al., 2002). Nitrate can also regulate the expression of several auxin-signaling genes including the *AFB3* auxin receptor (Vidal et al., 2010, 2013). It has been recently reported that nitrogen and small GTPase proteins act synergistically to regulate root hair growth in *Arabidopsis* (Bloch et al., 2011). In addition, it is known that auxin signaling pathway controls root hair growth (Lee and Cho, 2013). Based on these observations, we hypothesize that nitrate modulates root hair development by modulating auxin biosynthesis and signaling.

**Table 3:** **Ranking of the most consistent biological functions in the nitrate response**.

**GO**	**Description**	**Number of experiments**
GO:0000041	Transition metal ion transport	24
GO:0032787	Monocarboxylic acid metabolic process	23
GO:0015706	Nitrate transport	22
GO:0006007	Glucose catabolic process	21
GO:0006355	Regulation of transcription, DNA-dependent	21
GO:0019375	Galactolipid biosynthetic process	20
GO:0006569	Tryptophan catabolic process	19
GO:0006612	Protein targeting to membrane	19
GO:0006739	NADP metabolic process	19
GO:0009744	Response to sucrose stimulus	18
GO:0019344	Cysteine biosynthetic process	18
GO:0000165	MAPK cascade	17
GO:0010054	Trichoblast differentiation	16
GO:0016567	Protein ubiquitination	16
GO:0045893	Positive regulation of transcription, DNA-dependent	16
GO:0051973	Positive regulation of telomerase activity	16
GO:0006090	Pyruvate metabolic process	15
GO:0006499	N-terminal protein myristoylation	15
GO:0009694	Jasmonic acid metabolic process	15
GO:0043288	Apocarotenoid metabolic process	15
GO:0045892	Negative regulation of transcription, DNA-dependent	15
GO:0009687	Abscisic acid metabolic process	14
GO:0043623	Cellular protein complex assembly	14
GO:0051761	Sesquiterpene metabolic process	14
GO:0055080	Cation homeostasis	14

*GO terms with high semantic value (levels 7 and 8) and represented among genes regulated in more than half of the experiments analyzed are shown*.

### Weighted correlation gene networks predict functional modules in response to nitrate

**Figure 3:**
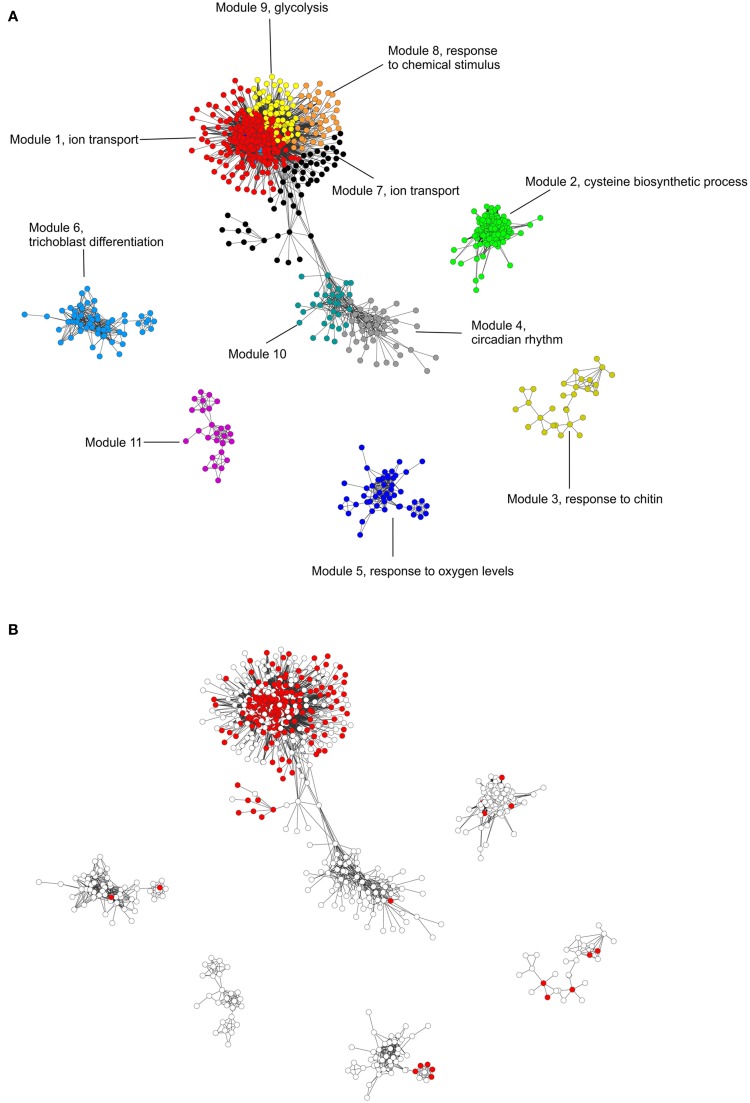
**Nitrate-responsive gene coexpression network. (A)** Colors are used to distinguish each gene network module. The most over-represented and consistent biological process GO terms (appear in at least 14 different experiments) are indicated in each module (File S2). **(B)** Red nodes indicate genes that respond similarly to nitrate treatments in NR-null mutants and wild-type plants indicating they respond directly to a nitrate signal (Wang et al., 2004). X and Y axes does not represent any particular scale (Table S3).

To analyze functional relationships among the 2286 nitrate responsive genes identified above we inferred a network as described previously (Langfelder and Horvath, 2008). In our analysis, 11 coexpression modules were identified and functional analysis indicated 9 out of the 11 modules had overrepresented biological functions. Interestingly, the gene network modules identified include robust functions of the nitrate response such as ion transport, carbon metabolism, response to chemical stimulus and trichoblast differentiation (Figure 3). The most consistent biological functions found associated to the nitrate response (Table 3) metal ion transport, monocarboxylic acid metabolic process, nitrate transport, glucose catabolic process or regulation of transcription are also overrepresented in these modules. Below we describe in more detail gene network modules 1, 6, 7, 8, and 9 containing these functions.

Module 1 is the largest module in the nitrate-responsive gene coexpression network, in terms of number of genes and number of connections. The top categories in this module are anion transport (GO:0006820, *p*-value = 2.60 × 10^−18^) and response to nitrate (GO:0010167, *p*-value = 7.75 × 10^−17^). Other biological functions involved in the nitrate response were also overrepresented, such as signaling (GO:0023052, *p*-value = 1.42 × 10^−7^) and regulation of transcription (GO:0006355, *p*-value = 8.54 × 10^−6^). Within the latter function, we found several families of transcription factors that were enriched, such as the G2-like or LBD discussed above.

Because hub genes play key functional roles in gene networks (Zotenko et al., 2008; Vidal et al., 2013), we identified those genes with the highest number of connections (degree) within each network module. The gene with highest degree in module 1 is an unknown HPP family protein (AT3G47980, pfam04982). HPP proteins are integral membrane proteins with four transmembrane helices. The identified protein has a predicted size of 27 kDa and a high pI (10.7) and based on his signal peptide sequence it is likely localized in the plastidic membrane. Another HPP family member (AT5G62720) has been identified in chloroplastidic membrane fractions by mass spectrometry (Ferro et al., 2010) and is also induced in response to nitrate. Using the eFP Browser (Winter et al., 2007), we found that the *HPP* genes are expressed in a tissue-specific manner (Figure S3). AT5G62720 expression is most abundant in photosynthetic tissues, while AT3G47980 is preferentially expressed in the roots suggesting the physiological roles of these two proteins may be different. Further research will be required to elucidate the role of HPP proteins in plants and their putative role in nitrate responses. However, because module 1 is enriched in transport functions, it is likely that highly connected genes such as the HPP genes identified may play a role in intracellular transport in the context of the nitrate response.

Enriched biological functions in module 7 are ion transport (GO:0006820, *p*-value = 1.73 × 10^−3^) and response to nitrate (GO:0010167, *p*-value = 2.46 × 10^−3^). These biological functions are also among the most enriched in gene network module 8. Genes of the CIPK family are among the most connected genes in both modules. CIPKs are Ser/Thr protein kinases that interact with calcineurin B-like calcium sensors and are involved environmental stress responses and nutrient sensing (Luan et al., 2009). Specifically, *CIPK3* is the second most connected gene in module 8 and *CIPK8* is the third most connected gene in module 7. *CIPK8* plays a role in regulation of gene expression of primary nitrate response genes (Hu et al., 2009). On the other hand, *CIPK3* has been widely analyzed in several experimental contexts, demonstrating its importance in plant development and adaptation to stress (Kim et al., 2003). However, its role in nitrate response has not yet been addressed. Interestingly, several protein phosphatases are also present in these modules, including PP2C (AT5G27930, AT5G26010, AT5G26010, AT3G16560, AT1G67820), dual phosphatase *ATPFA-DSP1* (AT1G05000), *AtMTM2* (AT5G04540), and *PP2A* (AT5G03470). With respect to the PP2A family, Heidari et al. (2011) showed that PP2A is required for the activation of nitrate reductase (NR). These results suggest the new kinases and protein phosphatases identified here may be important in phosphoproteome homeostasis for signaling and control of nitrogen responses in *Arabidopsis* roots.

Module 9 contains many overrepresented biological functions related to metabolism such as glycolysis (GO:0006096, *p*-value = 6.21 × 10^−9^), carboxylic acid metabolic process (GO:0019752, *p*-value = 1.12 × 10^−4^), hexose biosynthetic process (GO:0019319, *p*-value = 2.62 × 10^−3^), pyruvate metabolic process (GO:0006090, *p*-value = 5.85 × 10^−3^) and cellular nitrogen compound metabolic process (GO:0034641, *p*-value = 1.23 × 10^−3^). In addition, among the most connected genes we find several genes related to ammonium assimilation, such as glutamine synthetase (AT5G35630), glutamate synthase (AT5G53460), and isocitrate dehydrogenase (AT4G35260).

Finally, module 6 is a particularly interesting module in the context of nitrate responses because it is enriched in genes associated with trichoblast differentiation. Several genes within this module are essential for root hair development. One example is *Arabidopsis EXPANSIN 7* (*AtEXPA7)* gene, which is expressed specifically in root hairs (Lin et al., 2011a,b; Lan et al., 2013). The reduction of mRNA levels of *AtEXPA7* significantly affects root hair length (Lin et al., 2011a,b). Furthermore, it has been shown that this gene is able to complement a mutation in the rice *OsEXPA17* gene, suggesting functional conservation of root hair expansins in monocots and dicots (ZhiMing et al., 2011). Module 6 also includes the *AtXET14* gene, which encodes a xyloglucan endotransglucosylase enzyme implicated in root hair development (Maris et al., 2009). Adding purified recombinant *AtXET14* protein to MS medium for 2 days decreases growth of initiated root hairs and reduced the root elongation zone. In addition, *MRH6* and *COBL9* genes were also found in this module. These two genes were identified in a screening for root hair morphology mutants (Jones et al., 2006). Finally, peroxidase and extensin genes were over-represented in this module. Hydrogen peroxide is involved in root hair development (Dunand et al., 2007) and peroxidases are proposed candidate genes involved in this developmental process (Kwasniewski et al., 2013).

**Figure 4:**
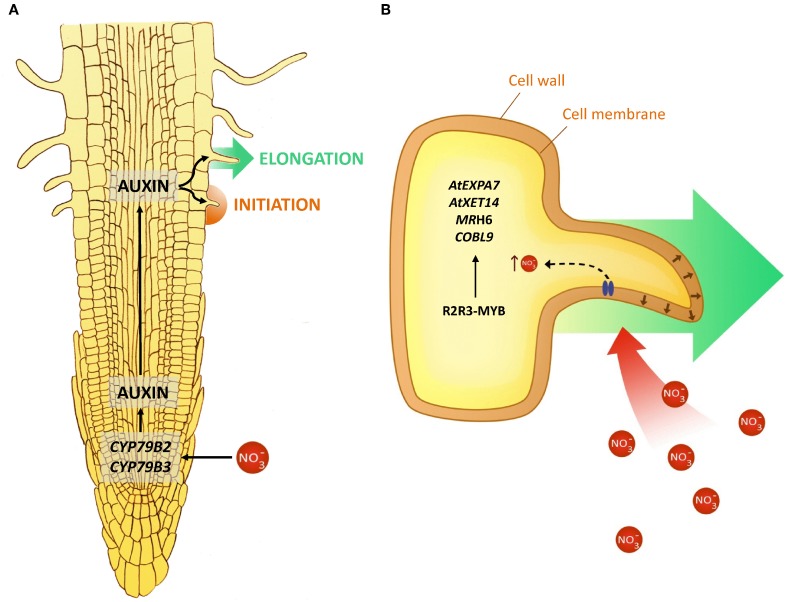
**Proposed model of how nitrate modulates root hair development in *Arabidopsis*. (A)** Nitrate treatments induce auxin biosynthesis and this phytohormone promotes root hair initiation and elongation. **(B)** Schematic detail of a root hair and genes involved in their growth mediated by nitrate.

Recently, an R2R3-MYB transcription factor was found to control development of root hairs in *Arabidopsis* (Slabaugh et al., 2011). We found five members of R2R3-MYB family (AT1G48000, AT5G14340, AT3G06490, AT5G60890, AT1G73410) within module 6. These genes belong to the only transcription factor family over-represented in the trichoblast differentiation network module, representing attractive candidate regulatory factors for root hair development in response to nitrate treatments in *Arabidopsis*. Figure 4 integrates these findings and proposes a simplified model for how nitrate may modulate root hair development. Nitrate is able to regulate expression of auxin biosynthesis genes. Auxin promotes initiation and growth of root hairs (Muday et al., 2012). It has been demonstrated that a significant amount of auxin is synthesized in the roots (Ljung et al., 2005), specifically in the apical region and *CYP79B2* and *CYP79B3* genes are involved in this localized auxin synthesis. Consistent with this model, we found nitrate induces expression of *CYP79B3* in 4 independent experiments (Gifford et al., 2008; Ruffel et al., 2011; Vidal et al., 2013 Álvarez et al., submitted) and *CYP79B2* in two different microarray experiments (Gifford et al., 2008; Vidal et al., 2013). Based on coexpression network analysis, we propose that these morphogenic changes would be mediated by cell wall proteins as extensins, expansins and peroxidases, which could be regulated by R2R3-MYB transcription factors (Figure 4B).

### Nitrate signals and not downstream products of nitrate reduction regulate genes found mainly in modules 1, 7, 8, and 9

Analysis of a NR-null mutant has shown that nitrate serves as a signal to control the expression of many genes in *Arabidopsis* (Wang et al., 2004). In order to distinguish nitrate-regulated modules vs. modules controlled by other N forms produced by nitrate reduction and assimilation, we integrated NR-null mutant transcriptome data (Wang et al., 2004) with our network analysis. As shown in Figure 3B, most of the nitrate-regulated genes are concentrated in central modules 1, 7, 8, and 9. Based on these results, transport, metabolism, and signaling biological functions represented in these network modules are robustly controlled by a nitrate signal. These results also suggest that biological functions such as circadian rhythms (module 4), response to oxygen levels (module 5), and trichoblast differentiation (module 6) are regulated by products of nitrate reduction. These results are consistent with previous findings such as the case of the master clock gene *CCA1* previously found to be regulated by organic nitrogen signals (Gutiérrez et al., 2008).

### Network analysis predicts central nitrate response modules are controlled by bZIP and Myb transcription factors

**Figure 5:**
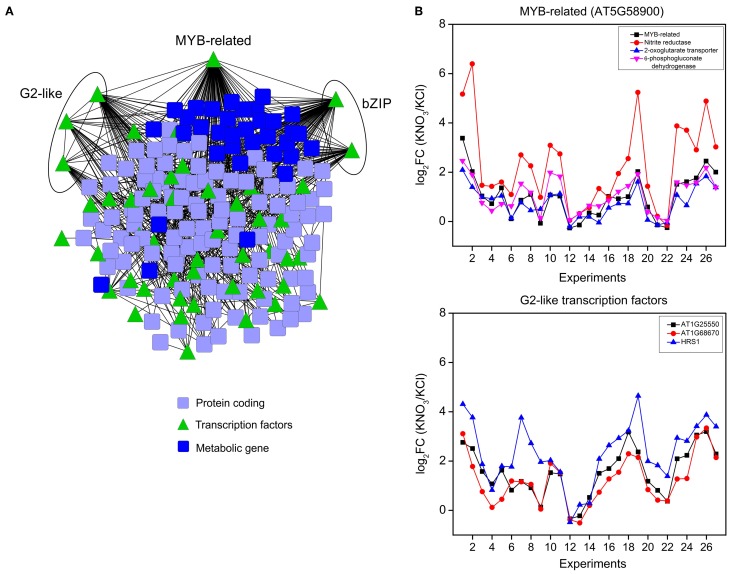
**Transcriptional control of the central modules in the nitrate gene network. (A)** A subnetwork of transcription factors and their potential targets derived from modules 1, 7, 8, and 9. Edges represent predicted regulatory interactions between transcription factors and target genes based on overrepresented transcription factors binding sites on the promoter of the targets. The most connected transcription factors are highlighted. **(B)** Expression patters of *MYB*-related transcription factor together with their three most correlated targets. Expression profiles of the three G2-like transcription factors are shown below.

To identify transcription factors that control essential and robust functions in the root nitrate response such as nitrate transport and assimilation, we focused in transcription factors from modules 1, 7, 8, and 9 and their possible targets. Figure 5 shows the subnetwork with edges between transcription factors and their putative targets taking into account the over-represented binding sites for the transcription factor in the promoter region of corresponding target genes using VirtualPlant software (Katari et al., 2010). In this network, MYB-related (AT5G58900) and bZIP (AT5G65210) genes showed the highest degree. Three different G2-like transcription factors (AT1G68670, AT1G25550, AT1G13300) were also found in top positions of the ranking of transcription factors with higher degree. As shown in Figure 5B, MYB-related gene coexpressed with nitrite reductase, 2-oxoglutarate/malate chloroplast transporter and a 6-phosphogluconate dehydrogenase gene from the oxidative pentose phosphate pathway. These results suggest this MYB-related factor controls basic aspects of nitrate metabolism, such as nitrate reduction, GS/GOGAT cycle and the generation of reducing equivalents.

A bZIP transcription factor identified as potential network driver (*TGA1*) belong to the subfamily TGA and another member of this family (*TGA4*) occupied the fifth position in the ranking of transcription factors with higher degree. These transcription factors have been implicated in bacterial defense responses. *tga1/tga4* double mutant plants show a greater susceptibility to infection by *Pseudomonas syringae* (Shearer et al., 2012). However, our analysis suggests that these transcription factors (*TGA1*, *TGA4*) could be important in the nitrate response of *Arabidopsis* roots. *TGA1* is the second gene of this subnetwork in terms of number of connections and it is differentially expressed in 9 different microarray experiments. *TGA1* is highly coexpressed with metabolic genes such as urophorphyrin methylase 1 and phosphoglucose isomerase 1. In fact, the biological function overrepresented with a lower *p*-value among the possible targets of *TGA1* is primary metabolic process (GO:0044238, *p*-value = 0.002). We have recently validated this hypothesis, demonstrating *TGA1* and *TGA4* transcription factors are important regulatory factors of the root response to nitrate treatments in *Arabidopsis thaliana* (Álvarez et al., submitted). Another transcription factor of the bZIP family implicated in N-responses is *bZIP11*. bZIP11 has been shown to regulate asparagine synthetase 1 and proline dehydrogenase 2 in *Arabidopsis* (Hanson et al., 2011).

G2-like transcription factors are members of the GARP superfamily and are characterized by a conserved domain (GARP) that is a single Myb-related DNA-binding domain (Sawaki et al., 2013). It is interesting to note that there are several connections between the three members of the G2-like transcription factors family, suggesting they respond to nitrate treatments in a coordinated fashion (Figure 5A). AT1G68670 is the G2-like transcription factor with the higher degree in this subnetwork and is coexpressed with other transcription factors such as another G2-like (Figure 5B), *ZINC FINGER PROTEIN 4* (AT1G66140) and MYB-related (AT5G58900). Moreover, because several protein kinases such as *MAPKKK16*, *WNK7*, and *CIPK1* are also present in this regulatory network, we hypothesize G2-like transcription factors are key regulatory genes involved in nitrate signaling leading to metabolic and developmental responses in *Arabidopsis* roots.

## Conclusions

Integrated network analysis of transcriptome data provided novel hypothesis about functions and regulatory mechanisms by which *Arabidopsis* plants respond to nitrate. Our meta-analysis better assessed the nitrate functional space than any single or integrated transcriptome study previously published. We estimated the mean functional coverage of any single experiment at about 31%. This result highlights the need for integrated data analysis to better map the functional space for any given perturbation. Moreover it underscores the need for using experiments carried out under non-redundant environmental conditions.

Our Systems approach identified nitrate regulation of root hairs as an important component of the plant developmental response to changes in N nutrition, a yet unexplored research area at the intersection of N nutrition and root biology. We provided concrete hypothesis for genes and connections among genes related to root hair differentiation in response to nitrate that have not been previously highlighted nor addressed experimentally. Our results also highlight the role of bZIP and G2-like transcription factors for regulation of important functions related to nitrate transport and signaling. G2-like transcription factors have not been characterized in the context of nitrate responses. Functional studies of these new candidate genes should help better understand regulatory mechanisms underlying root nitrate responses in *Arabidopsis* and other plants.

### Conflict of interest statement

The authors declare that the research was conducted in the absence of any commercial or financial relationships that could be construed as a potential conflict of interest.

## Supplementary material

Figure S1 **Controls performed to evaluate GO term robustness. (A)** Distribution of total and shared over-represented GO term levels between any combination of two experiments. To generate this graph we analyzed all possible combinations of two experiments. Level 1 is the most general GO term category and level 11 the most specific. The x-axis corresponds to the depth of a concept while the y-axis shows average number of GO terms for a given level. **(B)** Number of GO associated with each 2286 nitrate responsive genes or with 2286 randomly selected genes (average of 1000 iterations).

Figure S2 **Histogram of the percentage of genes associated with the shared GO terms between any combination of two experiments**.

Figure S3 **Gene expression patterns of HPP genes (AT3G47980 and AT5G62720) according to the *Arabidopsis* eFP browser**.

File S1 **Cytoscape file of the nitrate-responsive gene coexpression network**.

File S2 **Cytoscape file of the subnetwork present in Figure 5A**.

Table S1 **List of 2286 nitrate-responsive genes**.

Table S2 **Biological processes that are statistically enriched in response to nitrate**.

Table S3 **List of genes that were commonly expressed in nitrate reductase-null mutant and wild type**.
