# Supplementary figures and images for: Systems analysis of transcriptome data provides new hypotheses about Arabidopsis root response to nitrate treatments
Source: Front Plant Sci. 2014 Feb 7;5:22. doi: 10.3389/fpls.2014.00022 (PMC3917222; doi:10.3389/fpls.2014.00022)

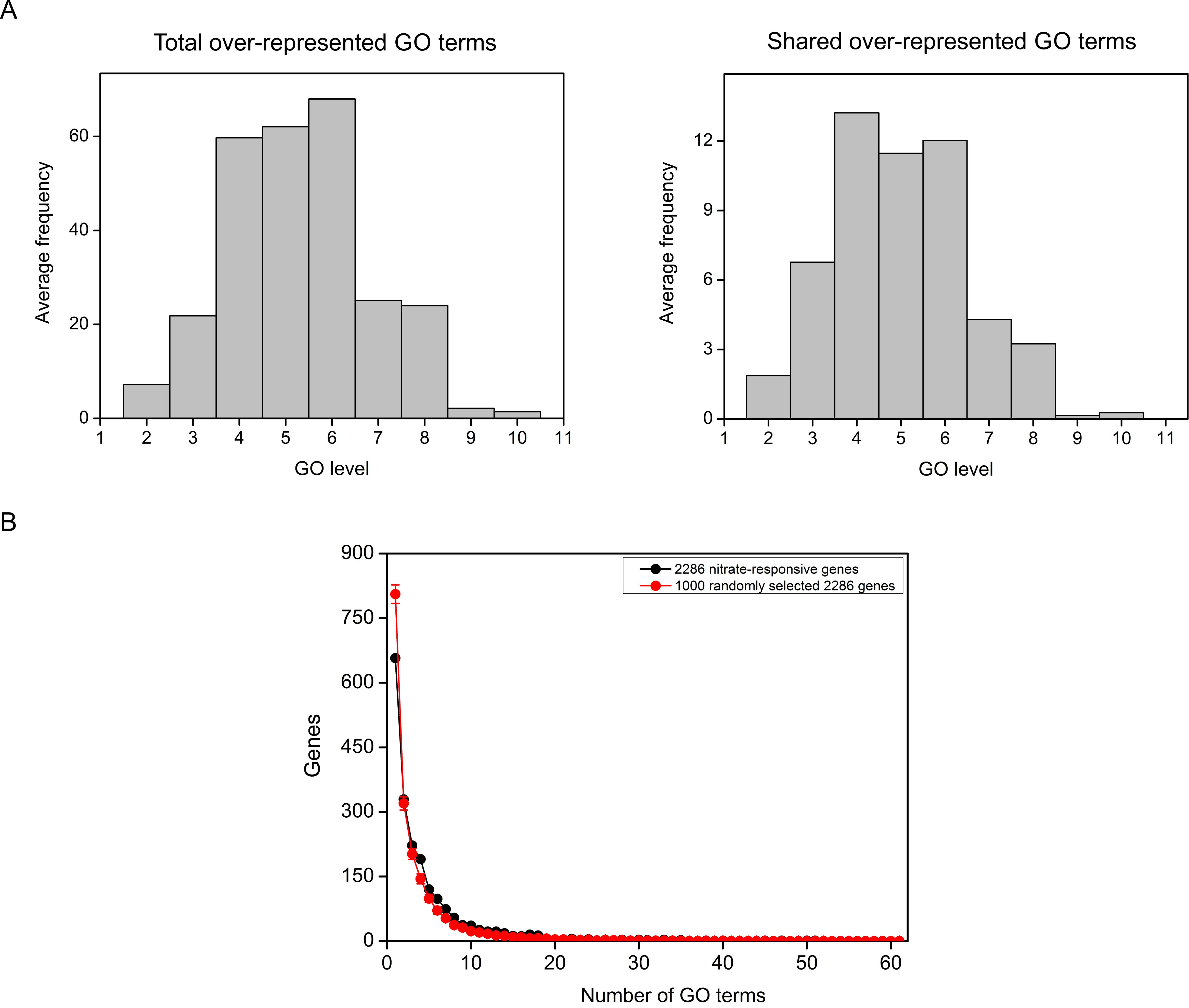

Supplement: Figure S1 — Controls performed to evaluate GO term robustness. (A) Distribution of total and shared over-represented GO term levels between any combination of two experiments. To generate this graph we analyzed all possible combinations of two experiments. Level 1 is the most general GO term category and level 11 the most specific. The x-axis corresponds to the depth of a concept while the y-axis shows average number of GO terms for a given level. (B) Number of GO associated with each 2286 nitrate responsive genes or with 2286 randomly selected genes (average of 1000 iterations). [file DataSheet1.ZIP › supplementary figures/Figure_S1.jpg]

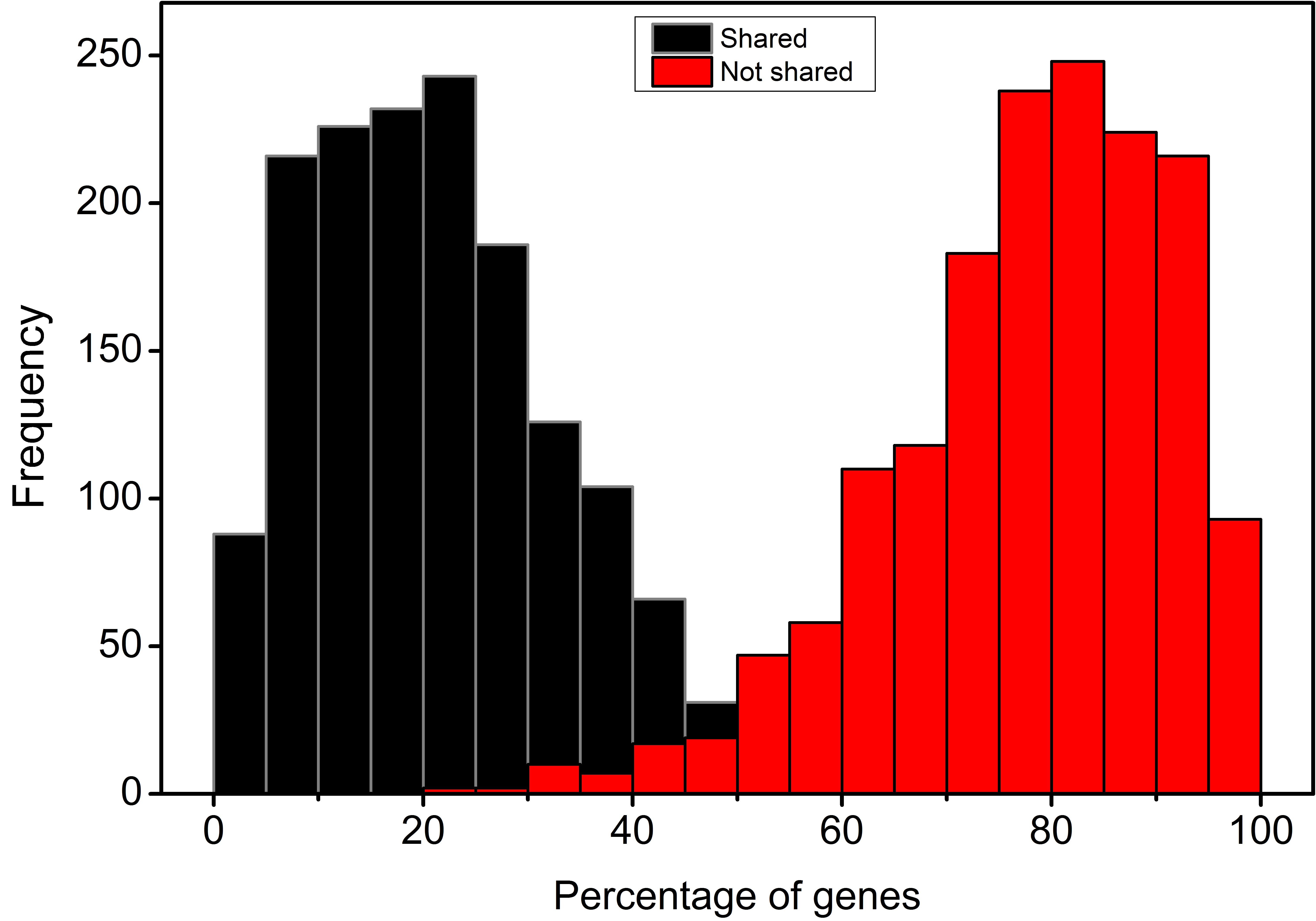

Supplement: Figure S1 — Controls performed to evaluate GO term robustness. (A) Distribution of total and shared over-represented GO term levels between any combination of two experiments. To generate this graph we analyzed all possible combinations of two experiments. Level 1 is the most general GO term category and level 11 the most specific. The x-axis corresponds to the depth of a concept while the y-axis shows average number of GO terms for a given level. (B) Number of GO associated with each 2286 nitrate responsive genes or with 2286 randomly selected genes (average of 1000 iterations). [file DataSheet1.ZIP › supplementary figures/Figure_S2.jpg]

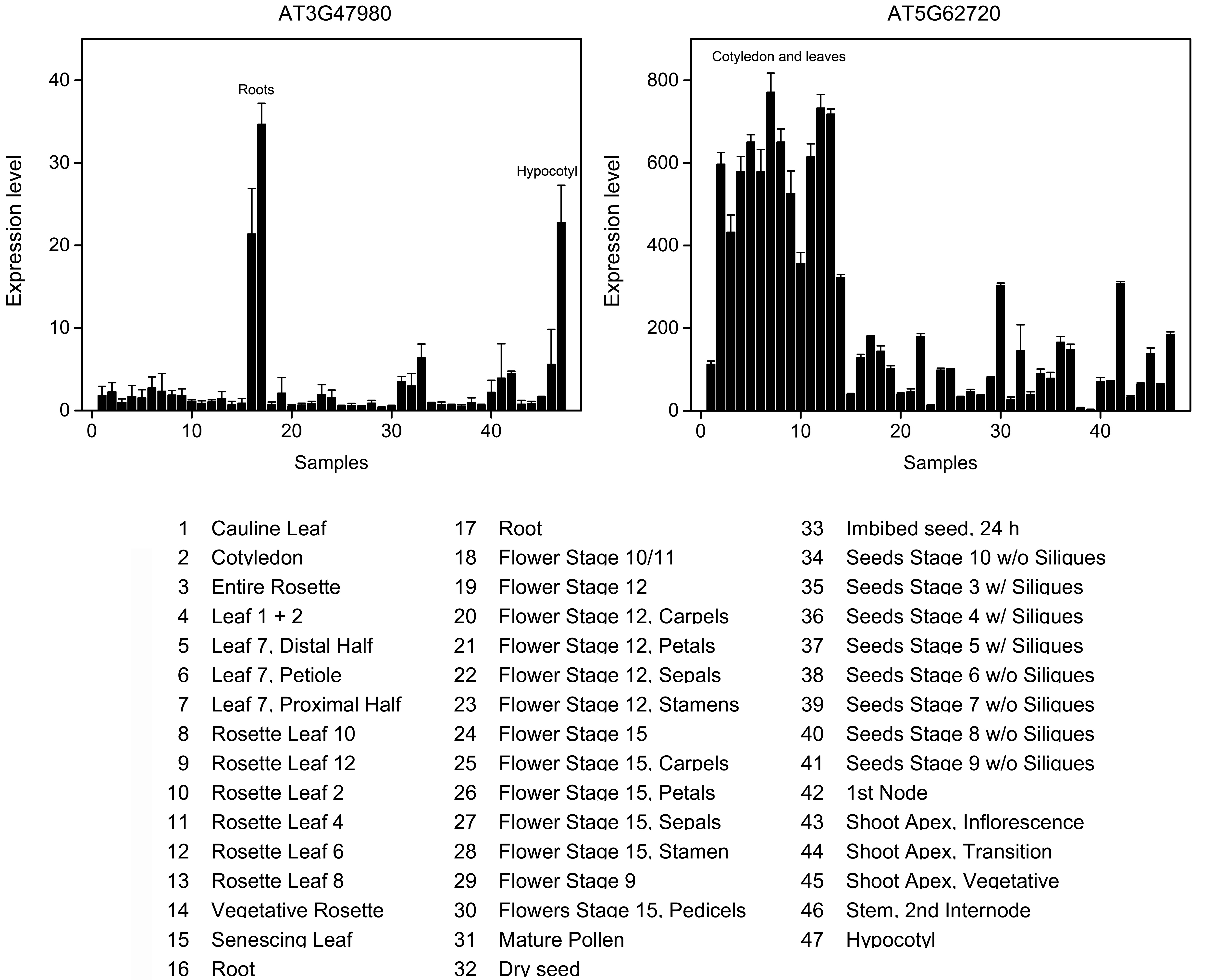

Supplement: Figure S1 — Controls performed to evaluate GO term robustness. (A) Distribution of total and shared over-represented GO term levels between any combination of two experiments. To generate this graph we analyzed all possible combinations of two experiments. Level 1 is the most general GO term category and level 11 the most specific. The x-axis corresponds to the depth of a concept while the y-axis shows average number of GO terms for a given level. (B) Number of GO associated with each 2286 nitrate responsive genes or with 2286 randomly selected genes (average of 1000 iterations). [file DataSheet1.ZIP › supplementary figures/Figure_S3.jpg]
